# Image Inpainting-Based Point Cloud Restoration for Enhancing Tactical Classification of Unmanned Surface Vehicles

**DOI:** 10.3390/s26051637

**Published:** 2026-03-05

**Authors:** Hyunjun Jeon, Eon-ho Lee, Jane Shin, Sejin Lee

**Affiliations:** 1 Department of Mechanical Engineering, Kongju National University, Cheonan 31080, Republic of Korea; 9598jhj@smail.kongju.ac.kr; 2 Unmanned System Team, Hanwha Systems, Gumi 39376, Republic of Korea; eonho810@hanwha.com; 3 Department of Mechanical and Aerospace Engineering, University of Florida, Gainesville, FL 32611, USA; jane.shin@ufl.edu; 4 Division of Mechanical & Automotive Engineering, Kongju National University, Cheonan 31080, Republic of Korea

**Keywords:** object classification, 3D point cloud data, LiDAR (light detection and ranging), surface vehicle, inpainting

## Abstract

The operational effectiveness of Unmanned Surface Vehicles (USVs) in modern naval scenarios depends on robust situational awareness. While LiDAR sensors are integral to 3D perception, their performance is frequently affected by incomplete data resulting from long-range sparsity and target occlusion. This study investigates a framework to restore incomplete point clouds to support improved surface vessel classification. The framework first estimates the target’s heading angle using a 2D area projection technique, combined with a descriptor to address orientation ambiguity. Subsequently, the 3D point cloud is converted into a 2D multi-channel image representation to leverage a deep learning-based image inpainting algorithm for data restoration. Finally, a high-density keypoint extraction method is applied to the completed point cloud to generate features for classification. This image-based approach is designed to prioritize computational efficiency and inference speed, facilitating deployment on resource-constrained maritime platforms. Experiments conducted on a simulator dataset reveal that the classification of restored point clouds yields higher accuracy compared to using the original, incomplete LiDAR data, particularly at extended distances (>70 m) and challenging aspect angles (0° and 180°). The results suggest the framework’s potential to address perception failures in sparse data scenarios, thereby supporting the operational envelope of USVs in contested environments.

## 1. Introduction

With the continuous advancement of LiDAR and other point cloud acquisition technologies, point clouds have established themselves as a core 3D data format across a wide range of industrial and research domains. In particular, they are utilized in various applications that require environmental perception, such as autonomous driving, map construction, and object recognition. More recently, the significance of LiDAR has also been emphasized in military and defense sectors. For example, LiDAR sensors mounted on unmanned systems, including autonomous combat vehicles, unmanned aerial vehicles (UAVs), and drones, are employed for 3D situational awareness, target detection, and object tracking on the battlefield. These sensors contribute to reliable perception capabilities even under low-visibility conditions.

A point cloud consists of a large set of 3D coordinates and can represent the shape, position, volume, and spatial structure of objects, making it suitable for reconstructing real-world environments into digital space. Owing to these properties, point clouds have become essential data sources in a wide range of computer vision and robotic perception tasks, including object classification [1,2,3,4,5,6], segmentation [2,4,7], pose estimation [8,9,10], and object detection [11,12,13,14]. In particular, their representational capacity and spatial resolution in complex 3D environments, where sensor input is often limited, enable precise object recognition and spatial understanding, which are often difficult to achieve with traditional 2D vision systems.

These technologies have recently been introduced into swarm USV, where they are increasingly employed. USVs require point cloud-based perception techniques to perform various tasks in maritime environments, including environmental monitoring [15,16], surveillance and reconnaissance [17] and autonomous navigation [18]. In particular, the distance information provided by LiDAR plays a role in preventing collisions among autonomously operating swarm vessels. Furthermore, object classification is required not only for detecting static obstacles such as buoys but also for identifying dynamic maritime targets, including fishing boats and cargo ships. In this context, the operational effectiveness of USVs in modern naval scenarios depends on robust and continuous situational awareness.

Marine environments may appear less intricate than terrestrial ones, yet USVs exhibit fundamentally different dynamic behaviors compared to ground robots. Unlike ground systems that can react immediately to obstacles, USVs typically have slower response times, making long-range object detection advantageous. To meet this requirement, maritime systems often adopt a multi-sensor approach that combines radar, cameras, and LiDAR for environmental perception [18,19]. Recent research has further emphasized that reliable environmental perception is essential for ASVs to perform diverse tasks safely, yet advancements have been slower than in the UGV domain due to the impact of waves and water reflections [20]. Radar provides long-range detection but suffers from limited spatial resolution, while cameras are hindered by sea fog and unsteady light intensity. LiDAR, on the other hand, provides highly accurate distance measurements; however, due to its radial scanning mechanism, its effectiveness is reduced at longer ranges because of line-of-sight constraints and decreased point density. As the observation distance increases, the number of points captured per unit area decreases significantly. Additionally, environmental factors such as surface reflections, sensor interference, and occlusion caused by surrounding structures often result in incomplete point cloud data, leading to the loss of critical 3D geometric information [18,21].

Due to these challenges, the utilization of LiDAR in maritime environments is inherently more limited than in terrestrial settings. In particular, point clouds acquired over water are often incomplete due to factors such as decreased point density with increasing distance, water surface reflections, and occlusions from surrounding structures. These deficiencies degrade the performance of downstream perception tasks, including object classification, segmentation, and detection. Therefore, effectively restoring incomplete point clouds plays a crucial role in enhancing the perceptual reliability of autonomous systems and contributes significantly to ensuring operational safety and robustness in real-world deployments.

This technical limitation poses a challenge to maritime operational doctrines. Strategic documents such as the U.S. Department of the Navy’s Unmanned Campaign Framework [22] emphasize the need for autonomous platforms capable of effective operation under incomplete information conditions. Consequently, the ability to ensure robust environmental perception even under degraded sensor data is fundamental to achieving reliable autonomous operations. In response to this demand, the present study investigates a point cloud completion approach designed to enhance classification performance. While native 3D point cloud completion methods often prioritize geometric fidelity, this study adopts an image-based inpainting strategy to optimize the balance between restoration accuracy and computational efficiency for real-time USV tasks. The results indicate that restoring the incomplete data consistently improves classification outcomes compared to processing the raw sensor output, particularly in scenarios where LiDAR points are sparse. The main contributions of this work are summarized as follows:

Pose Estimation: To ensure accurate completion of LiDAR point clouds, it is essential to estimate the orientation (heading direction) of the input point cloud in advance and perform a preprocessing step that aligns the data in a canonical direction. To achieve this, this study proposes a heading estimation algorithm for maritime vessels using a 2D area projection technique combined with the SPD^2^ descriptor.

Point-to-Image Conversion for Point Cloud Inpainting: In order to apply image inpainting algorithms, it is necessary to first convert the 3D point cloud into a 2D image. This study introduces a transformation method that faithfully preserves the geometric structure and depth information of the point cloud, enabling the reconstructed point cloud to achieve optimal performance in downstream classification tasks.

High-Density Feature Point Extraction: To maximize the classification performance using completed point clouds, we propose a high-density sampling-based feature point extraction method. Unlike sparse LiDAR observations from limited viewpoints, reconstructed point clouds encompass the object’s full geometry. The proposed method selects multiple representative points from structurally dense regions, enabling efficient and discriminative feature extraction for object classification.

## 2. Related Works

LiDAR-based point clouds acquired in real-world environments are often sparse or incomplete due to occlusions caused by objects or structures, decreased resolution at long ranges, and variability in surface reflectance. Such data degradation can lead to performance deterioration in downstream perception tasks, including classification, segmentation, and object detection. This issue becomes even more pronounced in open and dynamically changing environments, particularly in maritime domains. Consequently, recent studies have increasingly focused on point cloud inpainting as a technique to recover incomplete LiDAR data.

### 2.1. Point Cloud Inpainting

Point cloud inpainting, also referred to as point cloud completion, aims to reconstruct a complete point cloud from a partial input, typically acquired from a single viewpoint or under limited sensor coverage. Early methods relied on geometric priors, such as geometry-based completion [23,24], surface-oriented approaches [25,26], and symmetry-aware methods [9,27], which extrapolated missing shapes based on known patterns or regularities. However, these traditional methods struggled when dealing with large-scale occlusions or highly sparse inputs. With the advancement of deep learning, data-driven approaches have become the dominant trend in point cloud completion. For instance, PCN (Point Completion Network) [28] introduced an encoder–decoder framework capable of generating globally plausible 3D shapes from incomplete inputs. Subsequent models, such as TopNet [29], PF-Net [30], and SnowflakeNet [31], further refined this process using hierarchical decoding, fractal-like expansion, and Transformer-based refinement strategies to produce more detailed and semantically consistent completions.

### 2.2. Inpainting for Classification Accuracy Enhancement

Point cloud inpainting is gaining attention not only as a shape recovery technique but also as a means to enhance performance in downstream recognition tasks. By converting incomplete point clouds into semantically enriched and structurally complete data, the accuracy of classification or segmentation models can be enhanced. Recent studies have empirically demonstrated the benefits of this approach. For example, generative networks applied to aerial LiDAR data have been used to restore occluded or missing terrain information, leading to improved accuracy in semantic segmentation tasks [32]. Reconstructed point clouds provide more comprehensive representations, enabling classifiers or segmentation networks to better understand object structures and boundaries. Building upon these trends, the present study explores the effectiveness of image-based point cloud inpainting for LiDAR data restoration and its subsequent impact on object classification. The objective is not merely to enhance visual fidelity but to quantitatively assess whether inpainting contributes to measurable improvements in recognition performance using real-world LiDAR scenarios.

## 3. Methodology

### 3.1. Pose Estimation

Prior to reconstructing the point cloud, a preprocessing step is required to ensure accurate restoration. This section proposes a pose estimation algorithm that estimates the heading direction of a vessel point cloud and reorients the point cloud accordingly. The proposed algorithm computes the object’s orientation directly from the point cloud and represents it in the form of a bounding box. To construct the bounding box, information such as the object’s centroid coordinates (x, y, z), dimensions (length, width, height), azimuth, and heading angle is required. The centroid is calculated from the input point cluster, while the azimuth is derived based on the principal direction of the point distribution. The object dimensions are determined using prior results from LiDAR-based 3D object classification, allowing the bounding box to be appropriately scaled for the target class before the inpainting process.

#### 3.1.1. 2D Area Projection

**Figure 1:**
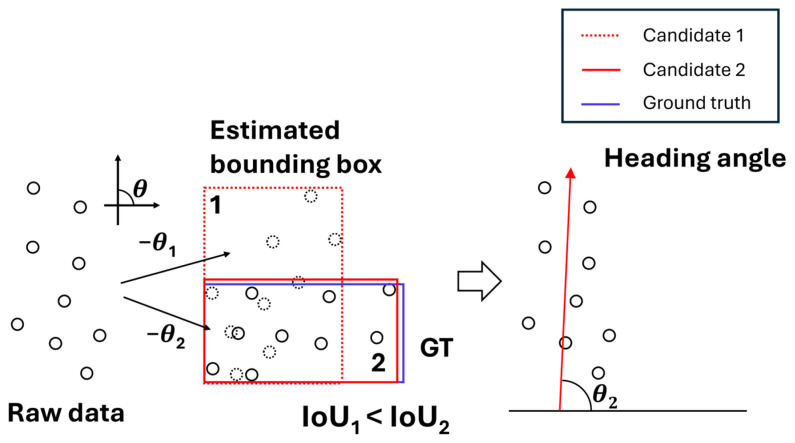
Illustration of 2D area projection method. The black coils represent the point cloud clusters, and the red arrow indicates the estimated heading angle.

The 2D area projection method estimates an object’s heading direction by projecting a 3D point cloud onto the XY-plane and searching for the optimal bounding box that best represents the shape of the resulting 2D distribution. As illustrated in Figure 1, the process involves incrementally rotating the point cloud around a reference center point. At each rotation angle, a bounding box is generated by calculating the minimum and maximum values along the X and Y axes. These bounding boxes are axis-aligned, meaning they remain rectangular and parallel to the X and Y axes, regardless of the rotation applied to the underlying point cloud. The rotation angle yielding the most compact or representative bounding box is then selected as the estimated heading direction of the object.

The generated bounding box is compared with a reference bounding box by calculating the Intersection over Union (IoU). The rotation angle that produces the bounding box with the highest IoU is selected as the vessel’s estimated heading angle. This study fundamentally assumes an operational military environment where target vessel types are standardized and their structural data is pre-collected. During this process, the point cloud is rotated at approximately 0.6° intervals over a 180° range. Under these conditions, the proposed heading estimation algorithm achieved a high orientation accuracy of 98.03% in simulator-based evaluations.

**Figure 2:**
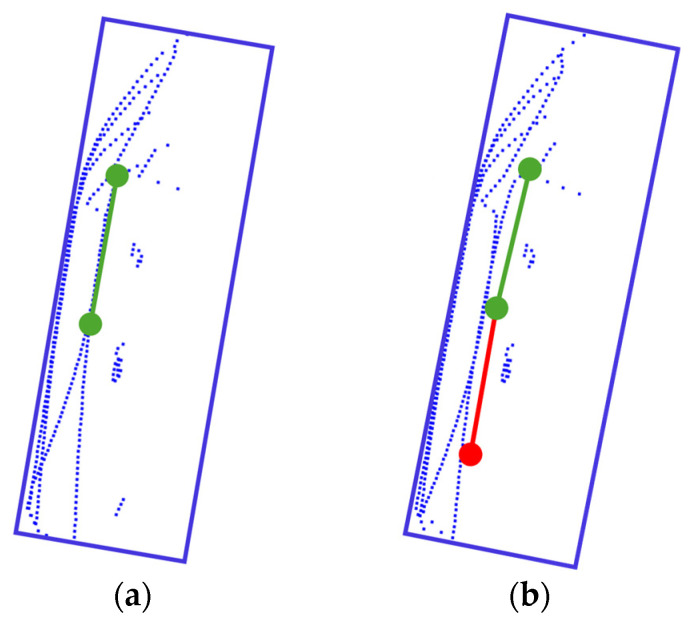
Results of the heading angle estimation for the ship (predicted bounding box is in blue, predicted heading angle in green, and ground truth heading angle in red): (**a**) Cases where the predicted heading angle matches the ground truth; (**b**) Cases where the predicted heading angle is in the opposite direction of the ground truth.

However, while this area projection-based approach can yield accurate estimations, for example, as shown in Figure 2a where the estimated heading aligns closely with the ground truth, there are also failure cases. Specifically, Figure 2b illustrates an example where the estimated angle is flipped by 180°, pointing in the exact opposite direction. To resolve this ambiguity and ensure consistency in estimation, the proposed method introduces a bow–stern discrimination strategy using the SPD^2^ descriptor to correct the heading angle.

#### 3.1.2. Bow–Stern Discrimination

It is inherently difficult to distinguish the bow and stern of an object using point cloud data alone due to its lack of semantic cues. To address this limitation, the present study employs the SPD^2^ [33] descriptor to estimate the vessel’s directional orientation. By learning the morphological characteristics of the point cloud, this descriptor enables semantic segmentation that identifies structural patterns unique to specific vessel parts, such as the wheelhouse, antennas, or other superstructures. Using these learned patterns, the system is able to infer the vessel’s forward-facing direction. As a result, the generated bounding box provides not only the spatial location of the object but also its precise orientation. This level of accurate localization is especially beneficial for real-time object recognition, autonomous navigation, and path planning in maritime environments.

### 3.2. Point to Image

In this study, an image inpainting technique [34] is employed for point cloud restoration. As a prerequisite, it is essential to convert 3D point cloud data into a 2D image representation. This point-to-image transformation is a critical step that directly influences not only the quality of the inpainting results but also the classification performance based on the restored point cloud.

Therefore, the image conversion process must be carefully designed to preserve the geometric structure and volumetric information of the point cloud as faithfully as possible, while also considering the post-inpainting completion rate. This study proposes a point cloud image representation method based on tripartite classification. The input point cloud is normalized using two different schemes and projected from two distinct viewpoints, resulting in four image generation configurations. Each of these configurations is subsequently evaluated by comparing both the inpainting outcomes and the classification performance of the restored point clouds, with the aim of identifying the most effective transformation method.

#### 3.2.1. 3D Point Cloud to 2D Image Conversion

The image inpainting algorithm employed in this study requires a fixed input resolution of 64 × 64 pixels. This resolution was selected to ensure optimal computational efficiency for real-time USV operations. Accordingly, it is essential to retain as much spatial information as possible within this limited resolution while projecting the 3D point cloud into a 2D image.

To achieve this, the 3D point cloud is orthographically projected onto a 2D plane based on a 64 × 64 grid. For each grid cell, the z-coordinates of the points are mapped to pixel values. However, a single-channel projection often fails to capture the vertical structural information of a vessel. To overcome this, this study proposes a tripartite layer mapping method that divides the point cloud into three horizontal layers along the *Z*-axis (height) and maps each to the RGB channels of an image. Specifically:

The Lower region (representing the hull) is mapped to the B (Blue) channel.

The Middle region (representing the deck and cabin structures) is mapped to the G (Green) channel.

The Upper region (representing masts and antennas) is mapped to the R (Red) channel.

**Figure 3:**
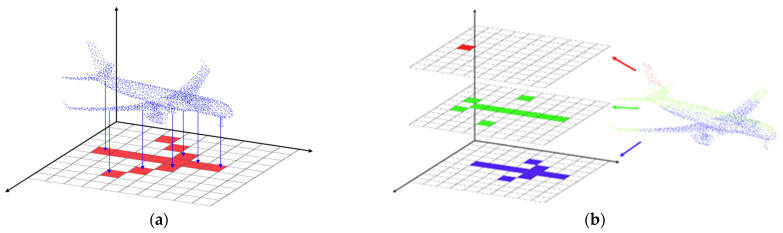
Illustration of Grid-based Point Cloud to Image Conversion Method: (**a**) Point Cloud to Image Conversion Process; (**b**) 3ch image Conversion Process. The red, green, and blue colors represent the height of the point cloud, categorized into three layers.

**Figure 4:**
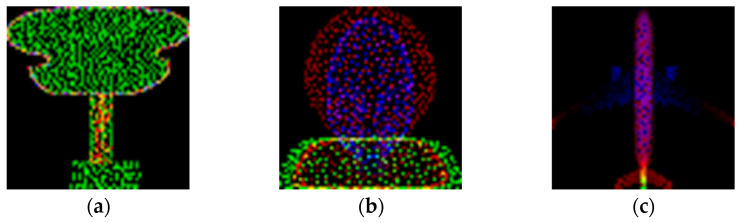
Generated 3-Channel Image Data: (**a**) Guitar; (**b**) Toilet; (**c**) Airplane

The z-values in each channel are normalized to a range of 1 to 255. This multi-channel approach allows the generative model to learn and restore the layered geometry of the vessel more effectively, as illustrated in Figure 3. The resulting examples of the generated 2D images are presented in Figure 4.

#### 3.2.2. Normalization of 3D Point Cloud

In this study, based on the previously proposed image transformation process, the input point cloud is normalized using two different strategies before image generation. These normalization methods affect the point density and depth representation in the converted images and serve as critical factors influencing the inpainting and reconstruction performance.

The first approach is global normalization, where the *x*, *y* and *z* values of the input point cloud are individually normalized to maintain a 1:1:1 ratio. This results in a uniformly scaled point cloud that preserves the overall shape without distortion, making it advantageous for retaining structural information in the generated image. However, because this method uses a wide spatial range when projecting the point cloud into a 2D image, the resulting point density can be low, which increases the potential for noise generation during the inpainting process.

The second approach is *Z*-axis normalization, which normalizes only the *z*-axis values of the input point cloud to fit within a 0~1 range, while preserving the original scale of the *x* and *y* axes. This technique enables the transformed image to maintain a visually faithful representation of the original shape and evenly encodes depth (volume) information across the RGB color channels. However, since only the *z*-axis is scaled, the overall image size becomes smaller, resulting in fewer points after reconstruction and ultimately leading to a lower completion rate.

The point cloud is converted into image representations using a combination of normalization schemes and viewpoints, resulting in four distinct image generation methods. Specifically, applying 1:1:1 normalization followed by top-view projection is referred to as Full_Top, while applying *Z*-axis normalization with a top-view projection is denoted as Z_Top. Similarly, applying 1:1:1 normalization with side-view projection is labeled Full_Side, and *Z*-axis normalization with side-view projection is termed Z_Side. These four methods (Full_Top, Full_Side, Z_Top, and Z_Side) are employed to effectively transform the point cloud into corresponding image formats.

### 3.3. Feature Point Extraction Based on High-Density Region Search

The reconstructed point cloud contains significantly richer geometric information compared to the original LiDAR data. To effectively utilize this enhanced data, it is critical to extract feature points that best represent the object’s structural characteristics. Unlike LiDAR scans collected from limited viewpoints, the reconstructed point cloud reflects the entire object shape, necessitating a feature extraction method capable of capturing comprehensive geometric patterns.

To this end, we propose a feature point extraction method based on high-density region search, which identifies regions within the point cloud where geometric information is densely concentrated. Specifically, the algorithm evaluates the local point density for each point, selecting feature points located in areas where a large number of neighboring points are clustered. This enables the extraction of representative features that emphasize structurally significant regions of the reconstructed point cloud. The selected high-density feature points are subsequently processed using the SPD^2^ descriptor, which transforms each feature point’s neighborhood into a structured feature image. These SPD^2^ images effectively visualize local geometric patterns within the point cloud. Using these feature images as input, a CNN-based classification network is trained to perform object classification on the reconstructed point cloud. This pipeline enables consistent feature representation across both original and reconstructed datasets, while also leveraging the structural richness of the inpainted data. The proposed framework thus facilitates a robust classification process that fully exploits the benefits of point cloud reconstruction.

#### 3.3.1. High-Density Feature Point Search Method

High-density feature points are extracted through the following procedure. First, for each point in the point cloud $P=\{p_{1},p_{2},\ldots ,p_{N}\}$, the number of neighboring points within a user-defined radius r is calculated. Using the Euclidean distance $\left||p_{j}-p_{i}|\right|$ between points, the neighborhood count $n_{i}$ for each point is defined as:

(1) $$n_{i}=\left|\left\{p_{j}\in P\setminus \{p_{i}\}\;|\;|p_{j}-p_{i}|<r\right\}\right|$$

This equation represents the number of neighboring points located within the radius r from the reference point $p_{i}$. After calculating all $n_{i}$ values, the points are sorted in descending order based on their neighbor count, and the top K high-density points are selected. However, each selected point $p_{k}$ must satisfy a spatial constraint such that it is at least a minimum distance $d_{\min}$ away from any previously selected high-density point $p_{k^{\prime }}$:

(2) $$|p_{k}-p_{k^{\prime }}|>d_{\min}\mathrm{for}\;\mathrm{all}\;\mathrm{previously}\;\mathrm{selected}\;p_{k^{\prime }}$$

**Figure 5:**
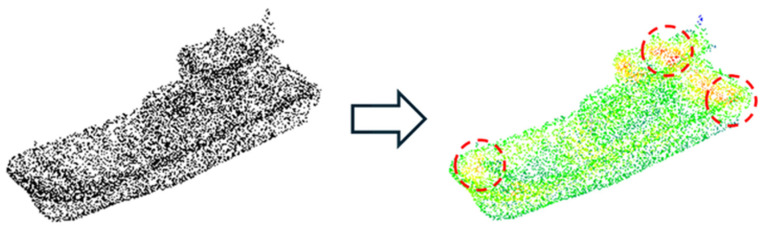
Visualization of high-density points identified through high-density region search. The red dotted circles indicate the locations of the identified high-density regions.

This condition prevents overlap between selected keypoints and ensures a well-distributed selection across the entire point cloud. The points selected by this method serve as representative locations centered around dense regions in the point cloud. These selected keypoints are visualized in Figure 5. These points provide meaningful features that can be utilized for identifying the shape or category of reconstructed objects. In this study, the selected points are used to generate feature images using the SPD^2^ descriptor, and the classification performance of the reconstructed point cloud is evaluated through a CNN-based classification algorithm.

#### 3.3.2. Classification Using SPD^2^-Based Feature Images

In this study, the classification framework for reconstructed vessel point clouds is di-vided into two main stages, specifically designed as a strategy to mitigate the domain shift between dense original data and restored data.

First, in the training stage, complete original point clouds are used as input to provide ideal geometric references. The rationale for utilizing the original dense data lies in the characteristics of the proposed ‘High-Density Feature Point Search’ method. Instead of training the model on a single global object representation, the algorithm identifies numerous high-density interest points across the entire vessel structure. For each of these points, a distinct 2D feature image is generated using the SPD^2^ descriptor and treated as an individual training sample. Consequently, the model learns a vast and diverse set of ideal local geometric features (such as specific structural patterns of masts, cabin edges, or hulls) rather than a single global representation per object. By learning these numerous ‘canonical’ signatures from original data, the model becomes capable of robustly identifying the essential geometric traits of each vessel class even in restored inputs.

For the classification task, standard CNN architecture was adopted to ensure an optimal balance between feature learning performance and rapid execution on re-source-constrained edge hardware. In the inference stage, the reconstructed point cloud is used as input. The top three dense points are selected, processed individually through the pre-trained standard CNN, and the final class is determined by averaging the confidence scores. This multi-point inference strategy further ensures that the framework remains robust to minor local artifacts that may exist in the restored regions.

## 4. Experiments

To verify the performance of the proposed algorithm, experiments were conducted using the ModelNet40 dataset [35] and a custom dataset collected through a self-developed simulator. ModelNet40 is a publicly available benchmark dataset consisting of 3D CAD models. It is widely used in studies related to 3D object recognition, classification, and reconstruction, as it includes various categories of general objects. The dataset comprises a total of 40 object classes, each consisting of dozens to hundreds of aligned 3D mesh models.

The simulator-based dataset was generated using a custom Unity-based simulator, which simulates the operating principles of an actual 64-channel LiDAR sensor to produce precise and realistic 3D point cloud data. Data collection was conducted on five selected ship models. Each model was placed at distances ranging from 30 m to 110 m in 10 m intervals and rotated in 1-degree increments over 360 degrees to collect point clouds. This experimental setup includes point cloud data generated under various observation distances and orientation conditions, enabling quantitative evaluation of the reconstruction and classification algorithms under different viewing conditions.

To quantitatively evaluate the alignment performance between the reconstructed point cloud and the original ground truth, three representative metrics were used: Chamfer Distance (CD) L1, Chamfer Distance (CD) L2, and F-score. These metrics are widely adopted in the field of point cloud completion and inpainting, as they effectively reflect both the overall distance-based similarity between points and the degree of local surface alignment.

### 4.1. Experimental Objectives and Setup

The experiments in this study were designed to evaluate the effectiveness of the proposed point cloud restoration and classification framework from multiple perspectives. The experiments comprise two main directions: quantitative performance evaluation and a comparison of classification accuracy before and after restoration.

#### 4.1.1. Experiment 1: Comparison of Restoration Performance According to Image Generation Methods

The first experiment was conducted based on the ModelNet40 dataset, with the primary objective of comparing and analyzing the impact of different image generation methods on restoration performance. For classification performance verification using the restored point cloud, this study selected a subset of ModelNet40 classes (Airplane, Guitar, Toilet) for experimentation.

**Figure 6:**
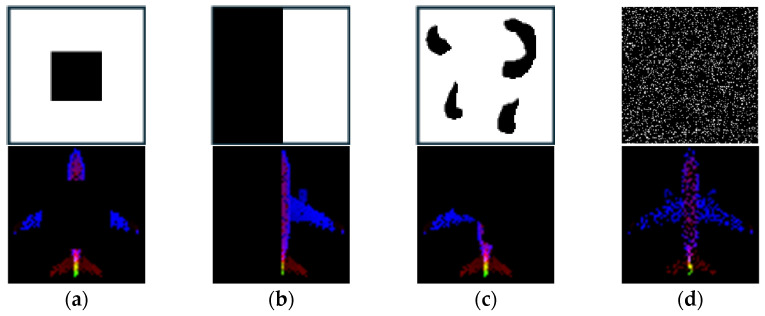
Examples of applied masks: (**a**) Stain; (**b**) Single-Side; (**c**) Center; (**d**) Random.

**Figure 7:**
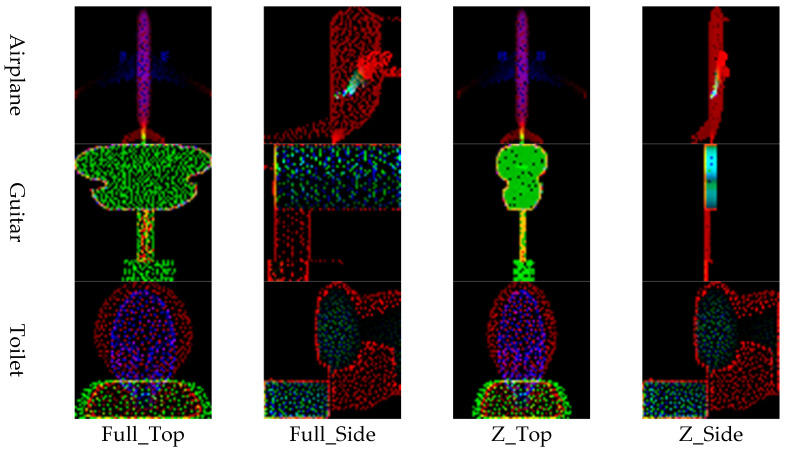
Experiment 2: Verification of LiDAR Classification Performance Improvement Using Simulator Data.

The input data for the restoration experiment were generated by applying four types of masks to intentionally remove or occlude parts of the point cloud. Examples of these masking methods and masked point clouds are shown in Figure 6. This approach simulates data loss scenarios that may occur during actual LiDAR-based observations. This paper proposes four image conversion methods, which are combinations of normalization methods and viewpoints, for transforming point clouds into 2D images. It compares how restoration quality varies depending on each method. Specifically, the combinations include Full Normalization and *Z*-axis Normalization, each applied to Top-View and Side-View perspectives, resulting in a total of four image generation combinations. Examples of input images generated by each conversion method are summarized in Figure 7, where the results for the Airplane, Guitar, and Toilet classes are visualized using Full_Top, Full_Side, Z_Top, and Z_Side transformations.

The same inpainting algorithm was applied to inputs generated by each image method, and the restored results were quantitatively evaluated using Chamfer Distance (CD) and F1-score. This analysis aimed to identify which image configuration yielded the highest restoration accuracy and geometric consistency, and to experimentally validate the effectiveness and justification of the proposed transformation methods.

#### 4.1.2. Experiment 2: Verification of LiDAR Classification Performance Improvement Using Simulator Data

The second experiment focuses on comparing classification performance before and after restoration, using a point cloud dataset of ships generated by a custom-built simulator. The purpose of this experiment is to verify whether point cloud restoration contributes to actual recognition performance improvement.

The classification models used in the experiment are structured as follows: For the pre-restoration point cloud, feature images generated based on the SPD^2^ descriptor were used as inputs to train the classification model. This approach aimed to evaluate classification performance using only the limited information contained in the original point cloud. For the restored point cloud, feature images were generated using the proposed high-density point selection-based feature extraction method. A separate classification model was then trained using these feature images, and the classification performance of the restored point cloud was evaluated. Through this comparative experiment, the quantitative impact of the proposed restoration method on actual object recognition and classification accuracy can be analyzed.

### 4.2. Results of Experiment 1

**Figure 8:**
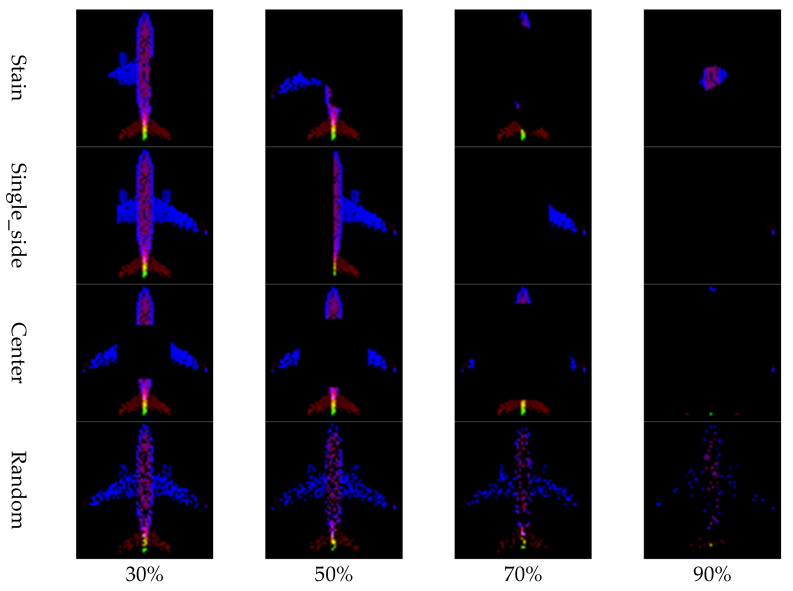
Occlusion patterns according to mask size by loss rate.

Experiment 1 was conducted based on the publicly available benchmark dataset ModelNet40, with the goal of qualitatively and quantitatively analyzing the impact of the four image generation methods proposed in this study on point cloud restoration performance. Three object classes (Airplane, Guitar, and Toilet) were selected from ModelNet40, and a total of 46 restoration experiments were conducted by applying various levels of data loss (30%, 50%, 70%, 90%) to each object. Figure 8.

#### 4.2.1. Visual Comparison

**Figure 9:**
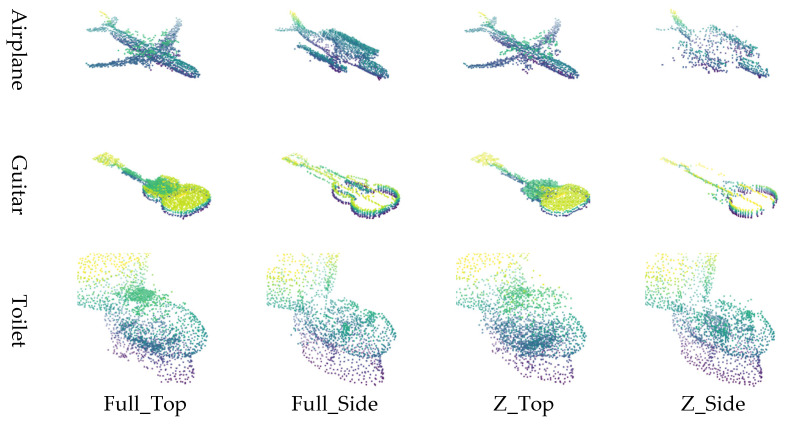
Reconstruction results with mask (Center_50%).

**Figure 10:**
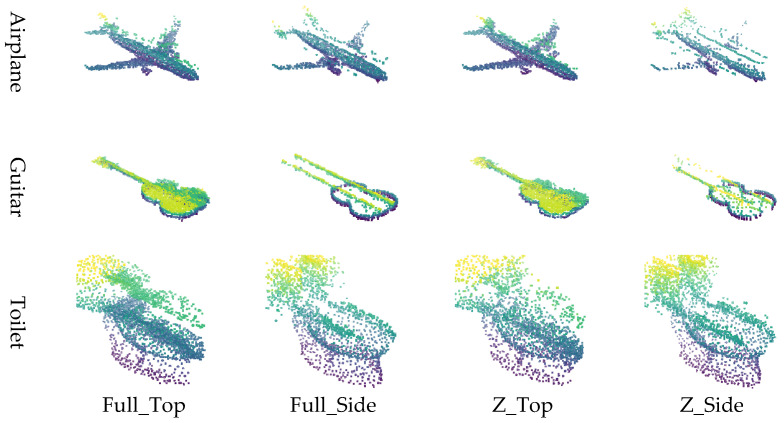
Reconstruction results with mask (Half_50%).

**Figure 11:**
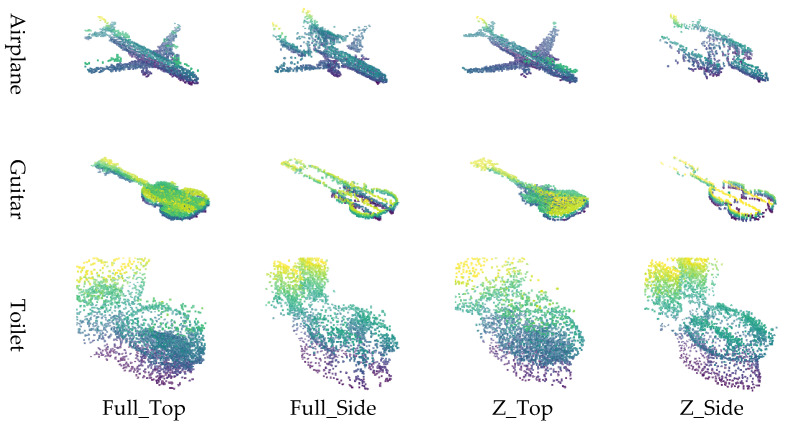
Reconstruction results with mask (Pattern_50%).

**Figure 12:**
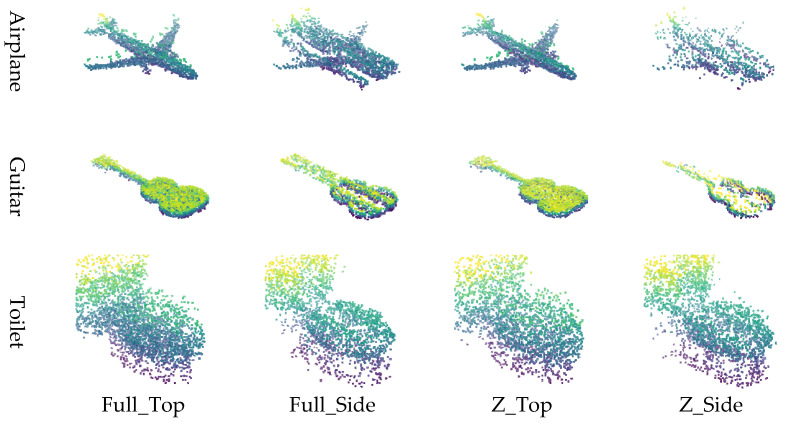
Reconstruction results with mask (Random_50%).

**Figure 13:**
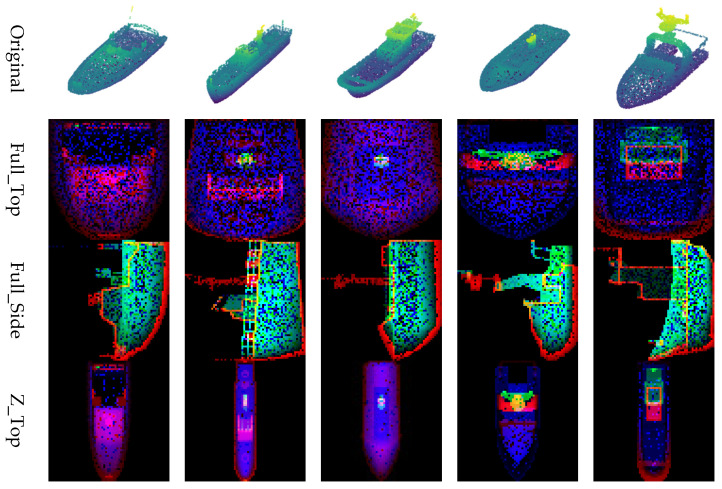

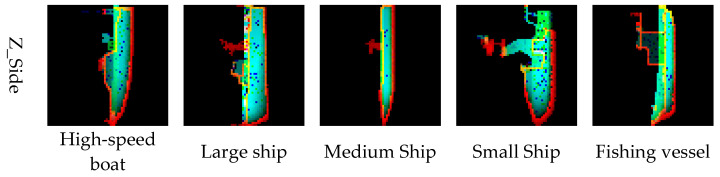
Image generation results by simulator ship type.

For visual comparison, four types of masks (Stain, Single_side, Center, Random) were applied to each object class (Airplane, Guitar, Toilet) at loss rates of 30%, 50%, 70%, and 90%, thereby inducing point cloud degradation. Representative visual analysis was conducted based on the restoration results with 50% data loss. Figure 9, Figure 10, Figure 11, Figure 12 and Figure 13 show the restoration results corresponding to each masking type (Stain, Single_side, Center, and Random) based on the four im-age generation methods proposed in this study (Full_Top, Full_Side, Z_Top, Z_Side). Through this visual comparison, the extent of structural restoration in the point cloud could be intuitively observed depending on the image generation method and the type of mask applied. In cases of successful restoration, the missing regions were naturally filled, and the object’s overall shape and geometric alignment were restored in a more coherent form.

#### 4.2.2. Quantitative Comparison

The quantitative evaluation in Experiment 1 was conducted using three representative metrics widely employed in the field of point cloud completion: Chamfer Distance-L1 (CD-L1), Chamfer Distance-L2 (CD-L2), and F-score. CD-L1 and CD-L2 measure the average point-wise distance between the restored point cloud and the ground truth. Lower values indicate higher restoration quality. CD-L1 reflects the overall point-wise error, while CD-L2 is more sensitive to outliers, capturing finer distortions in the restored point cloud. The F-score evaluates how accurately the restored point cloud overlaps the original within a predefined distance threshold. It is calculated as the harmonic mean of precision and recall, and higher values indicate better alignment. The results for each metric were averaged for the four proposed image generation methods and are summarized in Table 1.

#### 4.2.3. Quantitative Benchmarking

To evaluate the practical utility of our framework, we benchmarked it against existing algorithms, such as PCN and SnowflakeNet. While these models focus on high-fidelity restoration, our method prioritizes operational efficiency for real-time use. All experiments were conducted on a system equipped with an Intel Core i7-5930K CPU @ 3.50 GHz, 32 GB RAM, and an NVIDIA GeForce GTX 1080Ti GPU. As shown in Table 2, our approach shows a deficit in quantitative distance metrics compared to these heavy-duty architectures but offers a distinct advantage in restoration speed under the same hardware conditions.

### 4.3. Results of Experiment 2

This experiment focused on comparing object classification performance before and after restoration using a custom-built simulator-based ship point cloud dataset. The goal was to quantitatively analyze whether the proposed restoration method and the density-based key point classification framework contribute to actual improvements in recognition performance.

#### 4.3.1. Visual Comparison

**Figure 14:**
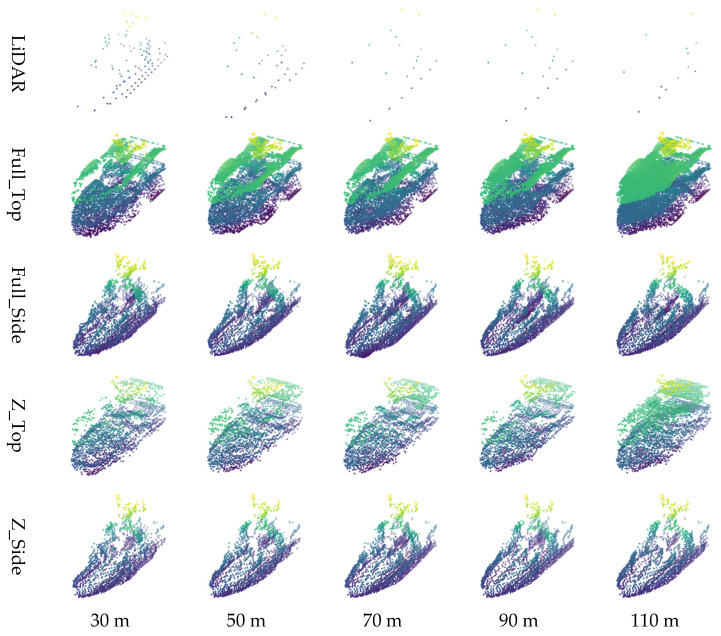
Point cloud restoration results by distance for simulator ships.

In Experiment 2, the Unity-based simulator dataset developed in-house was used to compare restoration performance by applying the four proposed image generation methods to five types of ship models. For each selected ship model, the original point cloud data was converted into 2D images using the four methods: Full_Top, Full_Side, Z_Top, and Z_Side. The conversion results for each ship model are summarized in Figure 14.

**Figure 15:**
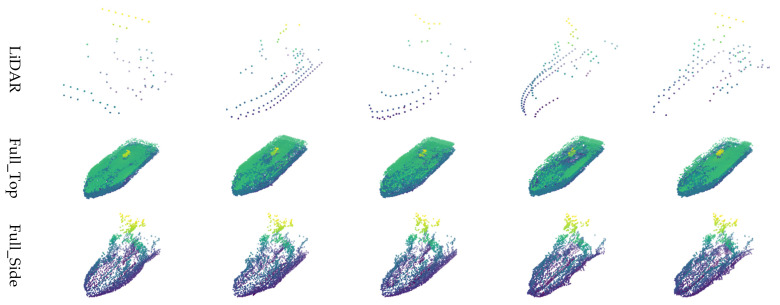

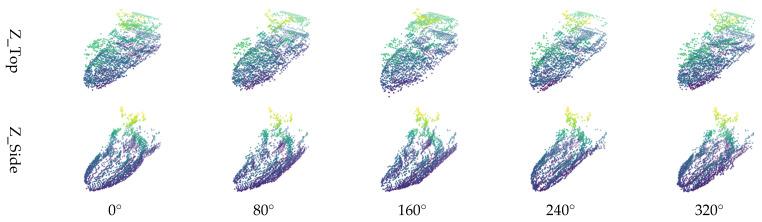
Point cloud restoration results by angle for simulator ships.

**Figure 16:**
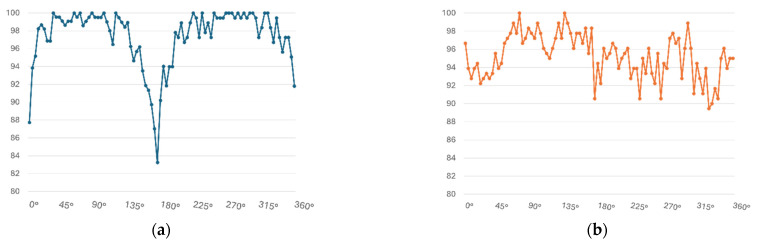
Classification Performance Graph by Angle Interval: (**a**) LiDAR Data Classification Results; (**b**) Reconstructed Point Cloud Classification Results.

For visual comparison of the restoration outcomes, one of the ships, referred to as the “Small Ship” model, was selected. Its restoration results were then visualized across varying observation distances (30–110 m) and rotation angles (0–320°). The results according to observation distance are shown in Figure 15, and those by rotation angle are presented in Figure 16. These visualizations provide intuitive insights into how the characteristics of LiDAR sensors, such as point cloud sparsity at longer distances and partial occlusion depending on viewpoint, affect restoration performance.

#### 4.3.2. Quantitative Comparison

The quantitative evaluation was conducted by comparing the classification results using both the original, unprocessed LiDAR point cloud and the restored point cloud. The experimental results are organized in Table 3 (by observation distance) and Table 4 (by observation angle). In terms of overall average accuracy, the classification model trained on the original LiDAR point cloud achieved the highest performance with an accuracy of 97.15%. In contrast, the models that used restored point clouds as input exhibited varying performance depending on the image generation method: Full_Top (95.23%), Full_Side (97.06%), Z_Top (90.18%), and Z_Side (96.20%). While the original LiDAR-based model showed a slight overall advantage, there were specific conditions based on distance and angle where the classification models using restored point clouds outperformed it.

In the distance-based evaluation, the classification accuracy of the original LiDAR model declined significantly beyond 60 m, due to the sharp decrease in point cloud density. However, the models using restored point clouds maintained relatively stable performance beyond 70 m, indicating that the proposed restoration method effectively mitigates performance degradation at longer distances. When comparing only the key range beyond 70 m, the accuracy of the original LiDAR-based model decreased to 95.17%, while the Full_Side method using the restored point cloud achieved 96.76%, showing an improvement of approximately 1.59%.

In the angle-based evaluation, when the ship was facing the LiDAR sensor frontally (0°) or from the rear (180°), the density of captured points significantly dropped due to the sensor’s structural limitations. This led to a decrease in accuracy for the original LiDAR-based classification model, with 95.26% at 0° and 91.51% at 180°. In contrast, the classification models using the restored point clouds benefited from structural completion and achieved higher accuracy under the same angle conditions. For example, the Full_Side method reached 96.89% at 0°, and the Z_Side method achieved 98.41% at 180°. These trends are visually depicted in Figure 16. Overall, this quantitative evaluation confirmed that the Z_Side method, based on *Z*-axis normalization and side-view image generation, produced the highest classification performance among the tested approaches.

#### 4.3.3. Practical Implications for Naval Operations

The implications of this study for naval operations lie not in the overall improvement in average classification accuracy but in the enhanced recognition performance at specific distances and orientations. In maritime surveillance missions, the extension of the maximum detection range (>70 m) enables earlier detection and more flexible evasive maneuvers, thereby enhancing situational awareness and operational responsiveness. Moreover, improved classification accuracy at 0° and 180° orientations contributes to consistent performance across all 360° azimuth angles, reinforcing the reliability and consistency of the classification algorithm. These improvements form a critical foundation for stable and continuous autonomous perception in naval operations, ultimately reducing the risk of missed or false detections during mission execution.

### 4.4. Ablation Study: Parameter Optimization

To determine the optimal parameters for the high-density feature point search, we analyzed the classification accuracy of the reconstructed point clouds by adjusting the search radius r and the minimum spatial distance $d_{\min}$. The results of this analysis are presented in Table 5. Both parameters were varied from 0.3 to 1.0.The highest classification performance was achieved at r = 0.5 and $d_{\min}$ = 0.5. If the radius r is too small, the algorithm fails to capture sufficient local geometric features, whereas an excessively large r incorporates information from an overly broad space, obscuring fine structural details. For the minimum distance $d_{\min}$, a value that is too small causes feature points to cluster in a single location, failing to represent the overall shape of the object. Conversely, an excessively large $d_{\min}$ reduces the total number of extracted points, leading to a loss of structural information.

## 5. Conclusions

This study proposed a framework for restoring incomplete LiDAR-based point clouds and effectively utilizing them for object classification. Centered around an image-based point cloud inpainting approach designed to improve restoration performance, the framework integrates four image generation methods that reflect the depth and geometry of the point cloud, a 2D area projection-based heading angle estimation algorithm for estimating object orientation, and a dense-point detection-based feature extraction method for CNN-based classification.

In Experiment 1, the restoration performance of the four proposed image generation methods was qualitatively and quantitatively evaluated using the ModelNet40 dataset. Visual analysis revealed that for objects such as Airplanes and Guitars, restoration performance degraded when using the side-view projection method. This is attributed to the fact that these objects possess thin structures along the *Z*-axis while being elongated along the X and Y axes; consequently, important structural features are lost during side-view projection due to insufficient spatial information. In contrast, the Toilet object, which exhibits a more balanced structure across all three axes, yielded stable restoration results in both top-view and side-view methods.

Quantitative analysis showed that the full normalization with top-view projection (Full_Top) achieved the best overall performance, followed by *Z*-axis top-view (Z_Top), full-side (Full_Side), and *Z*-axis side-view (Z_Side). These results indicate that top-view projection is generally more favorable for preserving an object’s geometric shape.

Finally, we benchmarked our framework against PCN and SnowflakeNet. While the quantitative restoration accuracy of the proposed method was lower than that of existing models, the proposed algorithm demonstrated faster restoration speeds. Notably, the processing speed using only a CPU was higher than that of the other algorithms. This highlights the practical feasibility of our algorithm for deployment on hardware-constrained platforms, such as USVs.

Experiment 2 focused on comparing classification performance before and after restoration using ship point cloud data generated with a Unity-based simulator. Visual analysis revealed that while Top-View restoration tended to introduce more noise, the Side-View methods suppressed noise more effectively, resulting in more coherent restorations. As distance from the object increased, the sparsity of the point cloud collected by the LiDAR increased, which in turn led to more noise in the restoration results.

Quantitatively, classification models using original LiDAR point clouds showed decreasing accuracy with increasing observation distance and varying angles, while models using restored point clouds maintained more stable performance. Notably, the effects of the proposed restoration method were most pronounced beyond 60 m and at angles of 0° and 180°. Interestingly, although Full_Top produced the best CD and F-score metrics in Experiment 1, Full_Side achieved the best classification accuracy in Experiment 2. This discrepancy can be explained by the interaction between the geometric properties of the data and the viewpoint used. As noted in Experiment 1, objects with balanced dimensions across the X, Y, and Z axes can be stably restored even with Side-View methods. The ship models used in Experiment 2 also had relatively balanced structures, allowing Side-View restorations to capture key structural features effectively.

Additionally, while Top-View methods tended to introduce more noise during restoration, resulting in degraded classification performance, Side-View methods produced more structured results, thereby contributing positively to classification accuracy. These experimental results support the practical feasibility of the proposed image-based point cloud restoration framework and suggest its potential for real-world applications, such as mitigating LiDAR data loss and improving object recognition performance. In particular, under conditions of partial data loss due to observation constraints, the proposed method can alleviate performance degradation and effectively recover structural information from various viewpoints, offering high practical applicability.

Based on the findings of this study, we propose two directions for future work. First, this study confirmed the potential of point cloud restoration to enhance the classification performance of USVs. Building on this, we plan to conduct practical validation using real-world vessel data in future research. Second, we are considering the integration of advanced generative models, such as Diffusion Models [36], which have recently gained significant attention in the field of point cloud completion. By incorporating such sophisticated models into our framework, we expect to achieve more accurate and structurally consistent reconstructions of vessel shapes.

## Figures and Tables

**Table 1:** Quantitative metrics comparison on the ModelNet40 dataset. L1 × 10^3^ and L2 ×10^4^ (lower is better). The best performance for each metric is highlighted in bold.

Generation Method	Metric		L1				L2				F-Score			
		Rate	30	50	70	90	30	50	70	90	30	50	70	90
	Model													
Full_Top	Guitar		2.69	4.7	7.87	10.8	1.6	2.73	4.83	7.07	97.1	94.81	91.86	89.47
	Toilet		22.9	43.27	59.55	70.23	27.81	47.73	69.47	87.26	85.49	69.9	59.13	53.01
	Airplane		5.56	8.85	15.9	18.5	4.04	6.07	11.38	13.45	93.46	89.18	81.35	78.21
	AVG		**22.56**				23.62				**81.91**			
Full_Side	Guitar		3.73	8.14	15.55	42.44	1.67	3.85	8.62	18.93	94.59	89.05	81.31	39.92
	Toilet		50.16	60.36	66.95	76.42	100.2	110.4	111.1	78.9	77.64	67.75	61.63	47.31
	Airplane		8.39	16.2	26.07	37.75	9.35	24.59	46.91	76.43	94.75	91.89	88.55	86.65
	AVG		34.34				49.24				76.75			
Z_Top	Guitar		3.51	9.87	12.55	19.49	1.58	5.56	6.98	12.75	95.3	88.92	85.79	80.95
	Toilet		30.01	55.38	62.68	102.9	35.37	68.82	70.87	12.58	77.74	63.67	55.91	39.24
	Airplane		4.89	9.95	14.74	19.78	2.95	6.9	10.19	14.15	93.95	87.7	81.92	76.22
	AVG		28.81				**20.72**				77.27			
Z_Side	Guitar		10.84	18.04	20.93	31.83	5.58	8.64	10.03	16.37	84.41	72.33	69.19	54.36
	Toilet		19.24	29.63	44.32	70.53	18.32	26.09	52.89	94.84	86.95	76.97	67.24	48.53
	Airplane		23.67	30.04	58.42	91.76	37.97	52.08	96.38	177.8	88.03	85.77	71.45	65.98
	AVG		37.43				49.74				72.60			

**Table 2:** Quantitative comparison of restoration performance and computational time between the proposed method and existing models.

Methods	PCN	SnowflakeNet	OUR
L1 Chamfer distance × 10^3^	9.64	7.21	23.56
L2 Chamfer distance × 10^4^	18.53	7.60	23.62
prediction Time (GPU) [s]	0.0012	0.021	0.0013
prediction Time (CPU) [s]	2		0.05

**Table 3:** Comparison of classification accuracy by distance between original LiDAR data and restored point cloud in the simulator ship dataset. Values representing higher accuracy than the LiDAR Classification in each distance interval are indicated in bold

Distance		30 m	40 m	50 m	60 m	70 m	80 m	90 m	100 m	110 m	Avg	Main AVG
LiDAR Classification		99.94	100.0	99.27	99.28	96.37	97.08	96.67	95.48	90.27	97.15	95.17
Restoration Classification	Full_Top	93.61	95.72	95.50	95.06	95.06	95.39	95.72	**96.61**	**94.44**	95.23	95.44
	Full_Side	97.50	98.22	97.33	96.72	**97.22**	**97.22**	95.56	**95.56**	**98.22**	97.06	**96.76**
	Z_Top	91.17	85.00	92.50	87.00	85.50	88.22	94.67	95.33	**92.22**	90.18	91.19
	Z_Side	96.72	97.61	95.72	93.28	**97.83**	**98.39**	**98.22**	91.78	**96.22**	96.20	96.49

**Table 4:** Comparison of Classification Accuracy by Angle between LiDAR Data and Restored Point Cloud in the Simulator Ship Dataset. Values representing higher accuracy than the LiDAR Classification in each orientation are indicated in bold.

Orientation		0°	45°	90°	135°	180°	225°	270°	315°	Avg
LiDAR Classification		95.26	97.82	98.22	98.02	91.51	98.12	99.72	98.62	97.16
Restoration Classification	Full_Top	94.32	95.41	97.54	97.63	**95.26**	94.17	94.86	92.84	95.25
	Full_Side	**96.89**	94.07	95.84	94.96	**97.28**	**99.21**	99.16	**99.11**	97.07
	Z_Top	88.49	87.51	89.13	91.46	89.23	91.95	93.68	89.93	90.17
	Z_Side	95.41	92.69	93.82	95.41	**98.42**	98.02	97.04	98.37	96.15

**Table 5:** Classification accuracy for different values of r and $d_{\min}$.

Parameter	r	$d_{\min}$	Accuracy	r	$d_{\min}$	Accuracy
Restoration Point cloud Classification	0.3	0.5	85.03	0.5	0.3	64.23
	0.5		96.76		0.5	96.76
	0.7		86.03		0.7	78.43
	1		75.23		1	74.23

## Data Availability

The data presented in this study are available on request from the corresponding author.
